# Myopericarditis Following the Third Dose of COVID-19 mRNA Vaccination: A Case Report

**DOI:** 10.7759/cureus.37005

**Published:** 2023-04-01

**Authors:** Aamir Saeed, Amman Yousaf, Muhammad Ahmad, Ghulam Mujtaba Ghumman, Ali Akram Khan

**Affiliations:** 1 Internal Medicine, Merit Health Wesley Hospital, Hattiesburg, USA; 2 Internal Medicine, McLaren Flint, Flint, USA; 3 Internal Medicine, St. Vincent Mercy Medical Center, Toledo, USA; 4 Cardiology, McLaren Flint, Flint, USA

**Keywords:** cardiac magnetic resonance imaging, covid-19 vaccination myocarditis, transthoracic echocardiogram, mrna-based vaccine, covid 19

## Abstract

Coronavirus disease 2019 (COVID-19) mRNA vaccine-related cases of pericarditis and myocarditis have been reported infrequently. Most of the patients usually present within a week of the vaccine, and on average, most of the cases were reported after the second dose of vaccine within two to four days. Chest pain was the most common presentation, and fever and shortness of breath were the other commonly reported symptoms. The patients can have positive cardiac markers and electrocardiogram (EKG) changes, and the cases can be mistaken for cardiac emergencies. We present a 17-year-old male patient with sudden onset substernal chest pain for two days who got the third dose of the Pfizer-BioNTech mRNA vaccine within 24 hours prior. EKG was remarkable for diffuse ST elevations, and troponins were elevated. Later, the cardiac magnetic resonance imaging confirmed the findings of myopericarditis. The patient was treated with colchicine and non-steroidal anti-inflammatory drugs (NSAIDs), completely recovered, and is doing fine to date. This case hights that post-vaccine myocarditis can be mistaken and early diagnosis and management can prevent unnecessary interventions.

## Introduction

The coronavirus disease 2019 disease (COVID-19) vaccine was approved to prevent COVID-19 in 2020 for adolescents ≥16 years [1]. To date, various local and systemic side effects have been reported in the literature. Local side effects include tenderness, redness, warmth, itching, rashes, and enlarged axillary lymph nodes that were self-limiting within one to two days. The most common systemic side effects include fever, myalgia, chills, fatigue, nausea, and anaphylaxis [1]. Myocarditis and pericarditis are infrequent side effects of the mRNA vaccine that were most commonly reported after the second dose of the vaccine and were more commonly reported in younger individuals [2]. In this article, we present a 17-year-old patient who presented with substernal chest pain within 24 hours of the third dose of the Pfizer-BioNTech vaccine and was diagnosed with myopericarditis.

## Case presentation

**Figure 1:**
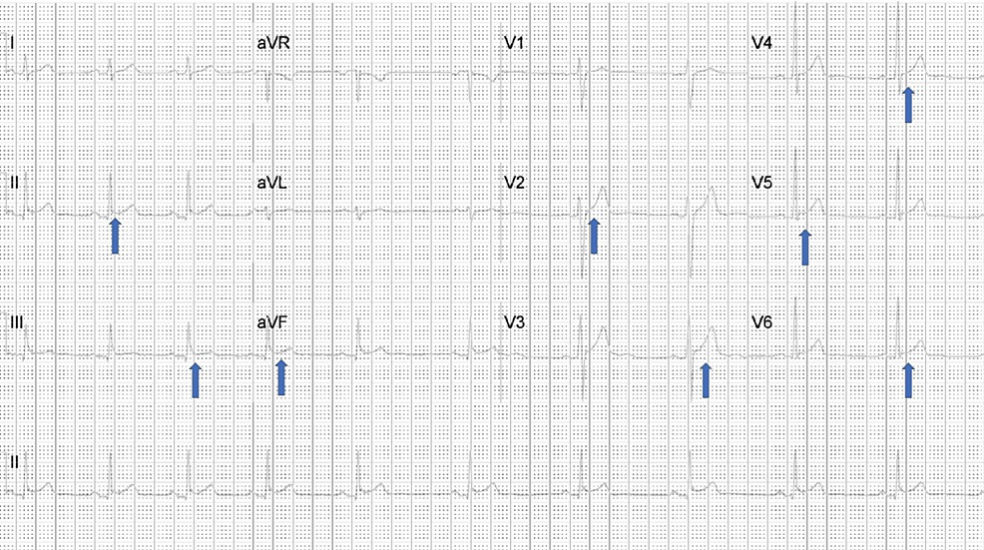
Twelve-lead electrocardiogram showing diffuse ST-elevations

A 17-year-old male patient with a past medical history of attention deficit hyperactive disease (ADHD) presented to the hospital with a two-day history of substernal chest pain. The pain was dull in nature, intermittent, 8/10 in intensity, non-radiating, and had no aggravating or relieving factors. The pain was associated with intermittent low-grade fever, rigors, chills, and shortness of breath. The patient received a third dose of the Pfizer-BioNTech COVID-19 vaccine within a day before he started to have chest pain. He previously denied having the COVID-19 vaccine and had no complications with the previous two doses of the same vaccine. His presenting vitals were blood pressure of 137/77 mmHg, heart rate of 120 beats per minute, oxygen saturation (SpO2) of 100%, respiratory rate of 18 breaths per minute, and temperature of 100.2° Fahrenheit. His cardiovascular examination did not reveal any murmurs or pericardial rub. Initial electrocardiogram (EKG) showed diffuse ST-elevations (Figure 1).

**Figure 2:**
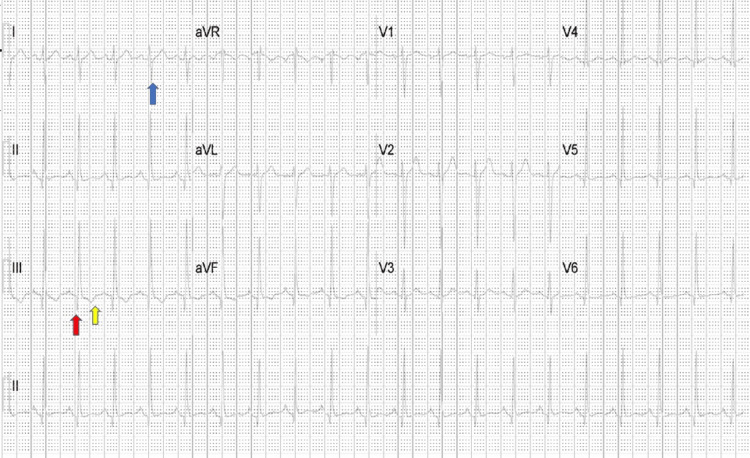
Twelve-lead electrocardiogram showing sinus tachycardia and the S1Q3T3 pattern

The patient was given high-dose antiplatelets and sublingual nitroglycerine, and cardiac enzymes, complete blood count, and metabolic profile were ordered. The patient's pain subsided; however, a repeat EKG showed sinus tachycardia and an S1Q3T3 pattern (Figure 2).

**Figure 3:**
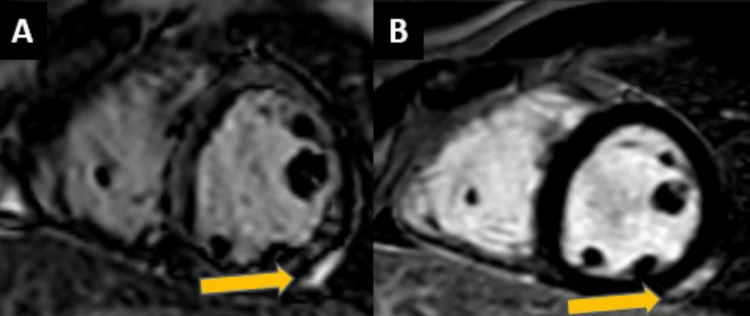
Gadolinium-enhanced MRI showing enhancement in the myocardium and adjacent pericardium.

The patient also underwent a CT chest with contrast, which showed no pulmonary embolism, and the lungs were unremarkable. Initial troponins were 0.03 ng/mL and 0.04 ng/mL, which trended up to 3.59 ng/mL within 24 hours of admission, and his C- reactive protein (CRP) was >300 mg/dL. Based on elevated inflammatory serum markers, EKG findings, and troponins, the diagnosis of pericarditis was made. The patient was empirically started on colchicine 0.6 mg twice a day and high-dose aspirin. His workup for the respiratory viral panel, COVID-19 polymerase chain reaction (PCR), and influenza were negative. On day two of admission, he underwent a transthoracic echocardiogram (TTE), which showed a normal left ventricular ejection fraction of 55%, and it showed no wall motion abnormalities or pericardial effusions. On day three of admission, the patient underwent gadolinium-enhanced magnetic resonance imaging, which demonstrated moderate subepicardial and mid-wall delayed enhancement in the inferolateral and basal inferior walls and areas of the adjacent pericardium. These findings were consistent with myopericarditis (Figure 3).

As the rest of his workup was negative and because of the recent vaccine, the vaccine was temporally considered the underlying cause of myopericarditis. The patient was discharged on seven days of colchicine and aspirin, and his symptoms completely resolved on day six after the presentation. He has had no other episodes of chest pain on a three-month follow-up and is doing well to date.

## Discussion

Various adverse events were reported after the second dose of the vaccine, including injection site pain, fatigue, myalgias, chills, arthralgia, fever, lymphadenopathy, headache, and myocarditis but rarely myopericarditis. Myopericarditis is a rare complication in adolescents after the COVID-19 mRNA vaccine. Most of the cases have been reported in men and after the booster dose of the vaccine. Though the exact pathophysiology has not been understood, inflammatory cytokines, unspecified immune response, or molecular mimicry between viral spike protein and unknown cardiac protein have been demonstrated to play a pivotal role in pathogenesis [3]. According to the US Centers for Disease Control and Prevention, myocarditis and myopericarditis rates are 12.6 cases per million among individuals between 12 and 39 years old after a second dose of mRNA vaccination. In these reported cases of myocarditis after the second dose of mRNA vaccination, young patients presented with chest pain and elevated troponin levels two to three days after the vaccination [4].

Patients usually present within two to four days after the second dose of the mRNA vaccine. Chest pain was the most common presentation, and other common symptoms were fever, cough, and shortness of breath [5,6]. Our patient also presented with chest pain and fever; however, he presented within 24 hours of the third dose of the COVID-19 vaccine. Moreover, the patients can have elevated inflammatory markers, positive troponins, and ST or T wave changes on EKGs, and in rare events, biventricular dysfunction has also been reported. Ambati et al. have recommended screening patients who present with chest pain, shortness of breath, or syncope within seven days of receiving the COVID-19 vaccine for myocarditis [2]. Elevated troponins and EKG changes should raise the suspicion of pericarditis or myocarditis in post-vaccine patients, and those individuals should get a baseline echocardiogram to evaluate the ventricular function, pericardial effusion, valvular abnormalities, or chamber enlargement.

Cardiac magnetic resonance (CMR) imaging has been widely used to diagnose post-vaccine myocarditis. The two main criteria for diagnosing myocarditis are the regional or global high T2-weighted signal intensity and late gadolinium enhancement on T1-weighted sequences [7,8]. The other supporting clues on MRI are pericardial effusion and regional or global wall motion abnormalities. Rosner et al. demonstrated CMR findings in seven patients with COVID-19 vaccine-related myocarditis. Patchy subpericardial, multifocal subepicardial, mid-myocardial, and basal anteroseptal mid-wall late gadolinium enhancement were the most commonly illustrated patterns on the post-contrast sequence, and three out of seven patients showed myocardial edema on T-weighted sequences. Our patient also showed moderate subepicardial and mid-wall delayed enhancement in the mid-inferolateral and basal inferior wall and areas of the adjacent pericardium. Nevertheless, histopathology is the gold standard for diagnosing myocarditis [9].

Myopericarditis is a self-limited inflammatory condition, and the outcome is usually a complete resolution of the symptoms. However, there are no treatment guidelines for COVID-19 vaccine-related myocarditis. Marshall et al. demonstrated using NSAIDs, intravenous immunoglobulins, oral prednisolone, and high-dose methylprednisolone in post-vaccine myocarditis [10]. Colchicine, beta-blockers, angiotensin-converting enzyme inhibitor, aspirin, and clopidogrel are the other medications that have been used [6]. The treatment was discontinued in most of the patients on the resolution of symptoms. Our patient was treated with colchicine and aspirin and had a complete resolution of symptoms within a week, and medications were discontinued.

## Conclusions

Myopericarditis is a rare complication of the mRNA COVID-19 vaccine, and clinicians should suspect myopericarditis in young patients with chest pain following COVID-19 mRNA vaccination. The patients usually present within a week of getting the vaccine, and early diagnosis can prevent unnecessary invasive procedures, especially in patients with chest pain, positive cardiac markers, and EKG changes. Cardiac magnetic resonance imaging is a very useful tool for early diagnosis. Though there are no guidelines for the treatment of vaccine-related myopericarditis, the outcome is usually good with conservative management. Further research is warranted to understand the pathophysiology and management of this rare complication.
